# Research agenda for antibiotic stewardship within the Veterans’ Health Administration, 2024–2028

**DOI:** 10.1017/ice.2024.6

**Published:** 2024-02-02

**Authors:** Daniel J. Livorsi, Westyn Branch-Elliman, Dimitri Drekonja, Kelly L. Echevarria, Margaret A. Fitzpatrick, Matthew Bidwell Goetz, Christopher J. Graber, Makoto M. Jones, Allison A. Kelly, Karl Madaras-Kelly, Daniel J. Morgan, Vanessa W. Stevens, Katie Suda, Barbara W. Trautner, Michael J. Ward, Robin L.P. Jump

**Affiliations:** 1 Center for Access and Delivery Research and Evaluation, Iowa City Veterans’ Affairs (VA) Health Care System, Iowa City, Iowa; 2 Division of Infectious Diseases, University of Iowa Carver College of Medicine, Iowa City, Iowa; 3 VA Boston Healthcare System, Department of Medicine, Section of Infectious Diseases. Boston, Massachusetts; 4 Harvard Medical School, Boston, Massachusetts; 5 Center for Care Delivery and Outcomes Research, Minneapolis VA Health Care System, Minneapolis, Minnesota; 6 Department of Medicine, University of Minnesota, Minneapolis, Minnesota; 7 VHA Pharmacy Benefits and Antimicrobial Stewardship Task Force, Department of Veterans’ Affairs, Washington, DC; 8 Center of Innovation for Veteran-Centered and Value-Driven Care, VA Eastern Colorado Healthcare System, Aurora, Colorado; 9 University of Colorado Anschutz Medical Campus, Aurora, Colorado; 10 VA Greater Los Angeles Healthcare System, Los Angeles, California; 11 David Geffen School of Medicine at the University of California, Los Angeles, California; 12 Informatics, Decision Enhancement, and Analytic Sciences (IDEAS) Center of Innovation, VA Salt Lake City Health Care System, Salt Lake City, Utah; 13 Division of Epidemiology, University of Utah School of Medicine, Salt Lake City, Utah; 14 Cincinnati Veterans’ Affairs Medical Center, Cincinnati, Ohio; 15 University of Cincinnati College of Medicine, Cincinnati, Ohio; 16 Boise Veterans’ Affairs Medical Center, Boise, Idaho; 17 Idaho State University, College of Pharmacy, Meridian, Idaho; 18 Department of Medicine, VA Maryland Healthcare System, Baltimore, Maryland; 19 Center for Innovation in Diagnosis, University of Maryland School of Medicine, Baltimore, Maryland; 20 Center for Health Equity Research and Promotion, VA Pittsburgh Healthcare System, Pittsburgh, Pennsylvania; 21 Division of General Internal Medicine, Department of Medicine, School of Medicine, University of Pittsburgh, Pittsburgh, Pennsylvania; 22 Center for Innovations in Quality, Effectiveness, and Safety (IQuESt), Michael E. DeBakey Veterans’ Affairs Medical Center, Houston, Texas; 23 Section of Health Services Research, Baylor College of Medicine, Houston, Texas; 24 Geriatric Research, Education, and Clinical Center (GRECC), VA Tennessee Valley Healthcare System, Nashville, Tennessee; 25 Department of Emergency Medicine and Department of Biomedical Informatics, Vanderbilt University Medical Center, Nashville Tennessee; 26 Technology Enhancing Cognition and Health Geriatric Research Education and Clinical Center (TECH-GRECC) at the VA Pittsburgh Healthcare System, Pittsburgh, Pennsylvania; 27 Division of Geriatric Medicine, Department of Medicine, School of Medicine, University of Pittsburgh, Pittsburgh, Pennsylvania

Antibiotic use is the strongest risk factor for the emergence and spread of antibiotic resistance, a global public health crisis.^1^ Mitigating this crisis must include efforts to measure and improve how antibiotics are being used in a variety of healthcare settings as well as in animals and agriculture.^2^ These types of improvement efforts are broadly described as antibiotic stewardship.

Approximately one-third of all antibiotic use occurs in human medicine; an estimated 30% of this human use is unnecessary.^3-5^ Among antibiotic use that is warranted, a substantial proportion has suboptimal antibiotic selection, dosing, or duration.^5^ To improve antibiotic prescribing, antibiotic stewardship programs are now mandated for all hospitals, nursing homes, and outpatient centers in the United States that are accredited by The Joint Commission or the Centers for Medicare and Medicaid Services.

Before these mandates went into effect, the Veterans’ Health Administration (VA), the largest integrated healthcare system in the United States, published a directive that required its medical centers to develop antibiotic stewardship programs.^6^ The VA National Antimicrobial Stewardship Task Force, established in 2011, continues to provide tools and resources that support effective antibiotic stewardship.^7^ The VA is a leader in stewardship implementation and stewardship-related research, with inherent advantages that include a motivated and engaged stewardship community, a robust health services research infrastructure, national-level organization of research, and the ability to synthesize and analyze national-level data through a central data repository, the VA Corporate Data Warehouse.

Since the publication of our last VA research agenda in 2018,^8^ VA researchers have informed the practice of antibiotic stewardship on a wide variety of topics, including the frequency of antibiotic-related harms, novel metrics for evaluating a hospital’s antibiotic use, clinical data on the optimal treatment of male urinary tract infections (UTIs), diagnostic stewardship of urine cultures, the diagnostic accuracy of hospital antibiograms in predicting the risk of antibiotic resistance, and approaches for providing more effective feedback to outpatient clinicians on their antibiotic-prescribing.^9-16^ To encourage further innovative and impactful research within VA and to support the national effort to improve antibiotic prescribing, we herein describe important knowledge gaps about the optimal use of antibiotics and the effective use of antibiotic stewardship strategies. The aim of this expert summary document is to provide a roadmap for the next 5 years of antibiotic stewardship investigations within and outside the VA.

## Guidance document development

Interested parties with relevant expertise across the VA were identified and recruited for participation (by D.J.L.). The 15 respondents, as coauthors, used an iterative process to identify research targets to include in this report. First, we presented the goals of this draft research agenda to the VA’s Antimicrobial Stewardship Taskforce’s monthly educational webinar audience (298 attendees) and the VA’s antimicrobial stewardship listserv (552 members) in May 2023. Using an electronic questionnaire, we sought feedback from these audiences about (1) which healthcare settings should be the focus of stewardship research and (2) which specific knowledge gaps are most important to address. In total, 40 attendees responded to this questionnaire. Second, we held virtual meetings of the coauthor group (May 1, 2023, May 18, 2023, July 7, 2023, and August 31, 2023) to discuss key knowledge gaps that should be highlighted.

To create an initial draft of the 20 most important research targets, 2 authors (D.J.L. and R.L.J.) combined feedback from these 2 sources. The coauthors achieved consensus through multiple rounds of written review and discussion, which included transforming the knowledge gaps into questions, ranking them from most to least important, and then considering those questions in the context of different healthcare settings: acute-care hospitals, outpatient care, long-term care (including spinal cord injury and disorders), periprocedural and surgical settings, dental clinics, and veterans receiving care in the community.

## Research agenda

Four research domains emerged during the synthesis and subsequent ranking of the knowledge gaps: stewardship strategies, clinical evidence, dissemination and implementation, and measurement (Table 1). Some questions applied to >1 domain and some were interconnected. The 3 highest-ranked research questions were prioritized for discussion below. We also addressed research questions specific to the different healthcare settings detailed above.

### Drivers of antibiotic resistance *[Question 1]*

Several facets of antibiotic use have been associated with the acquisition, emergence, and spread of antibiotic resistance, but it is unclear whether any of these aspects (eg, the use of specific antibiotic classes, prolonged duration of therapy, or suboptimal dosing) are disproportionately driving antibiotic resistance. Antibiotic de-escalation is a common target for inpatient stewardship activities. However, empirical data showing that changing from broad-spectrum to narrow-spectrum antibiotic agents reduces the likelihood of antibiotic resistance are limited. Gaining a better understanding of which aspects of antibiotic administration contribute to antibiotic resistance could inform the development and preferential implementation of stewardship strategies that address the major contributors to resistance [Question 7]. Currently, stewardship programs use an array of strategies to improve antibiotic use, including de-escalation, formulary restriction of broad-spectrum agents, and requiring justification for agents associated with increased risk for patient harm, such as fluoroquinolones. Knowing which of these activities is most effective at reducing selective pressure and other adverse events would help stewardship programs prioritize their limited resources and time.

### Antibiotic stewardship in non-hospital settings *[Question 5]*

Most antibiotic stewardship initiatives were developed to improve antibiotic use in acute-care hospitals. Unfortunately, inappropriate and unnecessary antibiotic-prescribing occurs across all healthcare settings. Although studies have described effective stewardship strategies in ambulatory care, emergency departments, and long-term care settings,^15,17-19^ real-world uptake of these strategies has been limited. Other settings, such as dialysis clinics or outpatient surgery centers, have been almost entirely absent from published stewardship interventions. Feasible, effective, and sustainable strategies for stewardship in non-hospital settings are important targets for additional research.

### Diagnostic stewardship *[Question 6]*

Diagnostic stewardship encompasses efforts to optimize the ordering of tests, the processing of laboratory specimens, and the presentation of test results to improve both diagnosis and treatment.^20,21^ Because the overdiagnosis of bacterial infection is a major driver of unnecessary antibiotic use, initiatives to improve diagnostic accuracy have potential to improve antibiotic use and patient care. Several promising strategies for stewardship of microbiologic tests, including urine and sputum cultures as well as *Clostridioides difficile* testing, have been published.^22-25^ End-user input will be important for developing additional strategies to influence how clinicians order tests and react to results, which may involve the use of clinical decision support tools [Question 12].^26,27^ Artificial intelligence could potentially improve clinicians’ ability to diagnose infections through a variety of mechanisms, such as by helping to generate a differential diagnosis, providing guidance on which diagnostic tests to order, and by assisting with test interpretation, although additional research is needed to determine how and when these technologies can be effective stewardship interventions [Question 8].

## Acute-care settings

The acute-care setting was an early target of VA antibiotic stewardship activities, and studies of several inpatient stewardship interventions have demonstrated tangible improvements in the selection, spectrum, duration, and proper dosing of antibiotic therapy.^7,10,28-30^ Meta-analyses and systemic reviews, including one from the VA Evidence Synthesis Program (ESP), have demonstrated that inpatient stewardship activities can safely improve antibiotic-prescribing and reduce antibiotic resistance.^31-33^ However, limited evidence is available to inform the staffing and information technology resources essential for the success of hospital-based antibiotic stewardship programs, including how these resources should vary by facility size and complexity. Contributing to this knowledge gap is a lack of detail on resources needed to implement successful interventions that have been described in the literature. Future implementation studies should quantify not only outcomes but also resources needed for implementation in an objective manner, including time, expertise and informatic resources [Question 10].

In hospitals that lack on-site infectious disease (ID) physicians and ID pharmacists, telehealth is a promising tool for stewardship, and the VA has specific expertise in how to use telehealth for this purpose.^34^ Strategies to advance the implementation and sustainment of telehealth-supported stewardship activities in resource-limited, acute-care settings are needed [Question 9].^34^

Future studies, preferably randomized-controlled clinical trials, could further inform several targets for acute-care stewardship activities. First, while oral antibiotic therapy is gaining greater acceptance for infections traditionally treated with parenteral therapy (eg, osteomyelitis), there is room to better define the optimal selection and dosing of oral agents [Question 3]. Second, more data on the comparative safety profiles of commonly prescribed antibiotic regimens would be valuable. A recent pragmatic trial comparing cefepime and piperacillin-tazobactam may serve as a viable model for future investigations.^35^ The VA Cooperative Studies Program research infrastructure and clinical trials networks have demonstrated success in the conduct of point-of-care and pragmatic clinical trials.^36-38^ Thus, the VA is an ideal setting for pursuing similar pragmatic trials designed to compare different standard-of-care antibiotic options. Third, large clinical trials could help identify specific situations where it is safe to decrease the length of recommended antibiotic therapy for common uncomplicated infections [Question 4]. For example, in cases of pneumonia, tools such as multiple polymerase chain reaction panels could improve identification of viral pneumonia and, in turn, identify patients in whom early discontinuation or avoidance of antibiotics is feasible.

One performance measure of acute-care antibiotic stewardship programs is the standardized antimicrobial administration ratio, which compares observed to expected antibiotic use at the ward and hospital-level without accounting for patient-level differences in case-mix across facilities. Although these types of gross measures may have a role in facilitating interhospital comparisons, more precise risk adjustment is necessary [Question 18]. Actionable metrics would be even more useful, such as those that quantify appropriate or optimal antibiotic use rather than overall use [Question 15]. To this end, there is a need for electronic quality indicators based on accepted clinical guidelines for common inpatient conditions (eg, pneumonia, skin and soft-tissue infection, and UTIs) [Question 20]. How these measures can be made valid, reliable, feasible, well calibrated, and interoperable across varying electronic health records, patient populations, and systems of care will be key challenges. Standardized, validated, patient-centered outcomes that measure the clinical impact of antibiotic stewardship activities would provide additional opportunities to monitor the effect of these programs, which may include reductions in length of stay and decreased hospital readmissions [Questions 14 and 16].

In summary, future antibiotic stewardship research in the acute-care setting should focus on (1) quantifying the amount of program resources necessary to achieve a measurable effect on antibiotic use; (2) building the evidence-base for using oral over parenteral therapy, comparing the safety of different antibiotic agents, and identifying opportunities to shorten duration of therapy for common infections; and (3) creating electronic quality indicators that measure guideline-concordant antibiotic use and patient-centered outcomes.

## Outpatient settings

Outpatient antibiotic stewardship encompasses efforts to measure and improve antibiotic-prescribing in primary care clinics, urgent-care clinics, emergency departments, and home-based primary care settings. Outpatient settings require different solutions than those used in acute-care hospitals due to different care models and workflows. Specific challenges to stewardship in these settings include (1) the time pressure faced by clinicians in emergency departments, urgent care, and primary care clinics; (2) competing priorities of patients and clinicians in a time-limited clinical encounter [Question 11]; and (3) the lack of accepted metrics that resonate with all stakeholders [Questions 14 and 15].

Findings from several systematic reviews, including the VA ESP, suggest that outpatient stewardship activities improve antibiotic-prescribing without short-term negative outcomes.^17^ Nevertheless, several knowledge gaps remain. First, few studies address the influence of outpatient stewardship activities on antibiotic resistance [Question 19] or on patient-specific outcomes [Question 14], such as antibiotic-related harm [Question 17] and microbiome effects [Question 2].^17^ Second, although studies have shown the efficacy of audit and feedback of prescription data, clinician focused education, and interventions embedded within the electronic health record (eg, order sets), which of these strategies is most effective in the outpatient setting remains unclear. Third, most outpatient stewardship studies have addressed antibiotic prescribing for acute respiratory tract infections and have not considered other common reasons for outpatient antibiotic-prescribing, such as for diarrhea, UTIs, or skin and soft-tissue infections. Fourth, both sustainability and scalability of outpatient stewardship activities have rarely been addressed.^17,39,40^ Finally, even within the VA, a pioneer in telehealth, data describing the influence of telehealth on outpatient antibiotic prescriptions are limited.^41^

Given these knowledge gaps, we propose the following areas for future antibiotic stewardship research in outpatient settings: (1) adapting strategies that apply the successes around antibiotic-prescribing for acute respiratory illnesses to other common outpatient infections, such as UTIs and skin and soft-tissue infections; (2) defining stewardship strategies that address the unique diagnostic and communication challenges posed by the increasing use of telehealth; (3) enhancing strategies to mitigate patients’ real and perceived antibiotic-seeking behavior for unnecessary indications; and (4) developing stewardship metrics that are tailored to and will resonate with clinicians practicing in outpatient settings.

## Long-term care and spinal cord injury units

Both residents in long-term care (LTC) facilities and persons with spinal cord injury/disorder (SCI/D) are special populations with a high frequency of antibiotic use and an increased risk for acquiring antibiotic-resistant organisms.^42,43^ Antibiotic decision-making in these populations is often complicated by difficulty ascertaining symptoms and limited access to diagnostic tests.

Although templates exist for establishing antibiotic stewardship programs in LTC, little guidance exists for SCI/D and evidence remains limited overall on optimal structure, strategies, implementation, and metrics.^44-46^ Most published antibiotic stewardship interventions in LTC have used education and guideline dissemination.^18,30,47^ The benefit of other stewardship strategies remains unclear in LTC, where the feasibility and effectiveness of any strategy may be hampered by lack of on-site ID or pharmacy expertise, lack of advanced diagnostic tests, and increased pressure from patients and caregivers to prescribe antibiotics [Question 11]. Telehealth offers great potential for more rapid and sustainable stewardship implementation in LTC facilities lacking on-site ID-trained physicians and pharmacists, but only a handful of telehealth studies have included LTC settings and none evaluated SCI/D populations.^48-50^ Only a few stewardship strategies have been evaluated specifically in SCI/D populations, with studies limited by small size and short follow-up periods.^51,52^

Nonlocalizing signs and symptoms are common in older adults as well as in persons with SCI/D. Vague signs and symptoms are common triggers for inappropriate antibiotic prescribing, particularly for suspected UTIs. Although guidance exists on which nonlocalizing signs and symptoms should prompt evaluation for infection and empiric antibiotics in LTC residents, effective implementation of these recommendations remains an ongoing challenge.^53-55^ Differentiation of asymptomatic bacteriuria (ASB) from UTI is especially challenging in people with SCI/D, who have both a high prevalence of ASB and frequent nonlocalizing symptoms and/or urinary symptoms that are not necessarily related to infection.^56,57^ Previous prospective research studies have demonstrated the value of standardized symptom assessments and infection definitions for ASB and UTI to support antibiotic stewardship in LTC.^25,30,58^ This work may serve as a foundation for similar prospective studies in people with SCI/D.

Given the dedicated and integrated care models for veterans in LTC and those with SCI/D, the VA is an ideal setting in which to study antibiotic stewardship for these populations. Future antibiotic stewardship research in LTC and SCI/D populations should (1) further evaluate the use of telehealth for implementing stewardship practices; (2) adapt traditional and newer stewardship metrics to LTC and SCI/D settings; and (3) develop validated tools to help distinguish ASB from UTI in patients with nonlocalizing symptoms.

## Other settings

### Periprocedural and surgical areas

Major evidence gaps for optimizing antibiotic use in procedural and surgical areas include the comparative effectiveness of different antibiotic agents for surgical prophylaxis, situations when surgical antibiotic prophylaxis can be replaced with other interventions to reduce infection risk, and the real-world effectiveness of weight-based antibiotic dosing and intraoperative redosing. Other knowledge gaps include limited data to inform reporting of direct clinical harms associated with unnecessary or suboptimal periprocedural and perioperative antibiotic use [Question 17]. Although professional guidelines recommend stopping antibiotic prophylaxis once the surgical incision is closed in the operating room, even in the presence of a drain, this guidance is frequently ignored and difficult to implement.^59,60^ Given the VA population of older men, there are abundant opportunities to deimplement this practice after urologic procedures.^61^

### Dental settings

Although professional dental associations support antibiotic stewardship in dental settings,^62,63^ there are few examples of successful programs.^64,65^ Even within the VA, 85% of antibiotics prescribed for prophylaxis by dentists are discordant with clinical treatment guidelines.^66,67^ Reasons for discordant antibiotic use include demands by patients and medical clinicians, social norms, and limited information on harms of short-term use of antibiotics for dental indications [Question 17].^68^ VA dentistry has been a leader within the profession on quality improvement, and this bodes well for research that seeks to incorporate antibiotic stewardship into the workflow of a dental encounter.

### Care in non-VA settings

Increasingly, VA patients are receiving care outside the VA system,^69^ though little is known about how the quality of antibiotic-prescribing for veterans compares between VA and non-VA settings. Non-VA care exemplifies common challenges for antibiotic stewardship activities outside traditional settings, including missing data elements and silos in health care.^70,71^ These information gaps present opportunities to advance existing knowledge while also informing and enhancing patient care.

A common type of non-VA care is outpatient hemodialysis for patients with end-stage renal disease. Dialysis centers prescribe antibiotics frequently; one study found that 1 in 3 patients received at least 1 intravenous antibiotic in US outpatient dialysis centers every year.^72^ Important knowledge gaps include the identification of specific targets for improved antibiotic prescribing in dialysis centers and the development of feasible, effective, and sustainable stewardship strategies to implement in these settings [Question 5].

Another common type of non-VA care is outpatient parenteral antibiotic therapy (OPAT), which is often managed by non-VA home care or infusion pharmacies and home health agencies. Because OPAT typically is not captured in VA data, it is unclear how OPAT is currently being used and how associated outcomes differ across facilities. If data capture could be improved, the VA may provide an opportunity to study different models for delivering OPAT and, in some circumstances, for deimplementing OPAT when oral antibiotics would be equally efficacious.

In conclusion, our multidisciplinary group of researchers and other interested parties have highlighted several important research targets for antibiotic stewardship. Research to address these targets could help improve the diagnosis and treatment of infections, optimize antibiotic stewardship activities, reduce antibiotic resistance, and improve the quality of healthcare both within and outside the VA.

## Figures and Tables

**Table 1.** Twenty Important Knowledge Gaps Related to Antibiotic Stewardship

Research Question	Acute-Care Hospitals	Outpatient Settings	LTC and SCI	Other Settings
**Clinical evidence**				
1. Drivers of antibiotic resistance: What are the comparative effects of different antibiotic agents, routes of administration, doses, and durations on the acquisition, emergence and transmission of antibiotic resistance?	X	X	X	X
2. How do antibiotics affect the microbiome, and which microbiome effects are detrimental to patients?	X	X	X	X
3. Which oral antibiotics are most efficacious in treating infections traditionally treated with parenteral therapy? What doses of these oral agents should be used?	X	X	X	
4. Are there specific situations when shorter than recommended antibiotic treatment courses for common uncomplicated infections are as efficacious as what is currently recommended? How can advanced testing methodology improve diagnostic accuracy and identify patients in whom early discontinuation of antibiotic therapy is safe?	X	X	X	
**Stewardship strategies**				
5. Antibiotic stewardship in nonhospital settings: Which stewardship activities are most feasible, effective, and sustainable in nonhospital settings, including postacute and long-term care, outpatient clinics, emergency departments, dialysis centers, and ambulatory surgery centers?		X	X	X
6. Diagnostic stewardship: Which approaches to diagnostic stewardship are most effective at improving antibiotic use, enhancing patient safety, reducing antibiotic resistance, and decreasing healthcare costs?	x	X	X	
7. Which antibiotic stewardship strategies are most effective at reducing the emergence, acquisition and transmission of antibiotic resistance?	X	X	X	X
8. How can artificial intelligence be leveraged to improve the diagnosis of infections, the prediction of antibiotic resistance, and the prescription of antibiotics?	X	X	X	X
**Dissemination and implementation**				
9. How can evidence-based antibiotic and diagnostic stewardship strategies be implemented and sustained across a variety of acute-care hospital settings to maintain effectiveness, including at hospitals without Infectious Disease (ID) physician and/or ID pharmacist expertise?	X			
10. What are the resources (eg, antibiotic stewardship expertise, administrative support, human resources, employee education, informatics, data visualization) needed to implement antibiotic stewardship initiatives that achieve a measurable impact on antibiotic use? How does that differ across healthcare settings?	X	X	X	X
11. How can antibiotic stewardship programs more effectively engage patients to be advocates for more judicious antibiotic use?	X	X	x	X
12. How can clinical decision support systems more effectively nudge prescribers to practice antibiotic stewardship and diagnostic stewardship?	X	X	X	X
13. How are stewardship data, goals and recommendations effectively communicated to prescribing clinicians and facility leadership? How should communication strategies be adapted based on the participants and the setting?	X	X	X	X
**Measurement**				
14. Which standardized, validated patient-centered outcomes should be measured to monitor the effect of stewardship on patient safety and quality of life? How should these vary among service lines (eg, general medicine, surgical services, other specialties) and across healthcare settings?	X	X	X	X
15. How can metrics and the dashboards used to display them be more actionable for antibiotic prescribers? How can metrics be leveraged to change antibiotic prescribing behavior?	X	X	X	X
16. What has been the effect of antibiotic stewardship programs on patient outcomes other than antibiotic use (eg, shorter length of stay, reduction in allergic reactions or drug–drug interactions)?	X	X		
17. How can antibiotic-related harms be quantified and electronically measured and then used to influence antibiotic prescribing behaviors?	X	X	X	X
18. How should stewardship metrics be risk adjusted to facilitate valid benchmarking across different clinicians, services, and locations?	X	X	X	
19. How can the effect of antibiotic stewardship activities on the prevalence of antibiotic resistance be accurately measured?	X	X	X	X
20. How can the quality of antibiotic prescribing be electronically evaluated across different clinicians, services, and locations?	X	X	X	X
